# Hydrogen sulfide promotes wheat immunity against stripe rust through TaATG6c persulfidation

**DOI:** 10.1007/s44154-026-00292-7

**Published:** 2026-02-13

**Authors:** Ying Ma, Fangfang Peng, Ran Zhao, YuPing Li, Jiayu Qian, Yizhe Wang, Guozhi Sun, Jiayu Meng, Jisheng Li, Zhensheng Kang, Xiaojing Wang

**Affiliations:** 1 College of Life Sciences and State Key Laboratory for Crop Stress Resistance and High-Efficiency Production, Northwest A&F University, Yangling, 712100 Shaanxi China; 2 College of Natural Resources and Environment and State Key Laboratory for Crop Stress Resistance and High-Efficiency Production, Northwest A&F University, Yangling, 712100 Shaanxi China; 3 College of Landscape Architecture and Arts and State Key Laboratory for Crop Stress Resistance and High-Efficiency Production, Northwest A&F University, Yangling, 712100 Shaanxi China; 4 College of Plant Protection and State Key Laboratory for Crop Stress Resistance and High-Efficiency Production, Northwest A&F University, Yangling, 712100 Shaanxi China

**Keywords:** Hydrogen sulfide, Wheat stripe rust, Autophagy, TaATG6c, Persulfidation

## Abstract

Hydrogen sulfide (H_2_S) is a key gaseous signaling molecule involved in plant growth and stress responses, yet its role in wheat resistance to stripe rust remains poorly understood. Here, we show that exogenous H_2_S enhances resistance of wheat (*Triticum aestivum* L.) to *Puccinia striiformis* f. sp*. tritici* (*Pst*), the causative agent of stripe rust. Comparative persulfidation proteomics identified the autophagy-related protein TaATG6c as a *Pst*-responsive H₂S target. Site-specific mass spectrometry and a modified biotin-switch assay demonstrated that Cys177 and Cys180 of TaATG6c undergo H₂S-induced persulfidation. Structural modeling based on AlphaFold predicted that these two site mutations reduced the binding activity of ATG6c to ATG14. Functional characterization using virus-induced gene silencing (VIGS) revealed that TaATG6 positively regulates wheat immunity against *Pst*, as silencing *TaATG6* promoted fungal growth. Moreover, *TaATG6* expression was markedly induced during *Pst* infection. Notably, the resistance-promoting effect of NaHS was compromised in *TaATG6*-silenced plants. Conversely, transient overexpression of *TaATG6* enhanced wheat resistance to stripe rust, whereas mutation of Cys177 and Cys180 attenuated this effect. Endogenous biotin-switch assays further showed that TaATG6c persulfidation exhibits pathogen-responsive and dynamic characteristics, which were abolished in the *TaATG6*^C177A/C180A^ mutant. Consistently, H₂S treatment and *Pst* infection stimulated the accumulation of lipidated ATG8 (ATG8–PE), indicating activation of autophagy, while this response was largely abolished in *TaATG6*-silenced plants. Together, these results suggest that H₂S promotes autophagy initiation through persulfidation of TaATG6c, thereby enhancing wheat resistance to stripe rust and highlighting a redox-regulated mechanism underlying plant stress adaptation.

**Supplementary Information:**

The online version contains supplementary material available at 10.1007/s44154-026-00292-7.

## Introduction

Wheat (*Triticum aestivum* L.) is the most important cereal crop worldwide and a Wheat (*Triticum aestivum* L.) is the most important cereal crop worldwide and a cornerstone of global food security. In recent years, wheat yields have been severely compromised by various diseases, among which stripe rust, caused by the obligate biotrophic fungus *Puccinia striiformis* f. sp*. tritici* (*Pst*), poses one of the most serious threats to global wheat production. Under favorable environmental conditions, this disease can lead to yield losses of up to 60% (Bandral et al. 2025). The highly dynamic nature of *Pst* populations presents a significant challenge to the effective prevention and control of stripe rust (Zhang et al. 2022). To address this persistent threat, it is essential to exploit resistance from traditional wheat germplasm, while effector identification provides valuable molecular insights into *Pst* pathogenicity and host–pathogen interactions (Tian et al. 2024).

Autophagy is a highly conserved process that maintains cellular homeostasis and restricts pathogen invasion by targeting intracellular pathogens and effectors for degradation. In *Arabidopsis*, the selective autophagy receptor NBR1 mediates the removal of viral capsid proteins, thereby contributing to antiviral defense (Qi et al. 2021). Beyond direct pathogen degradation, autophagy also modulates plant immunity by regulating immune signaling, programmed cell death (PCD), and reactive oxygen species (ROS) production (Marshall and Vierstra 2018). In wheat, autophagy has been implicated in resistance to stripe rust, with *Pst* infection inducing the expression of the autophagy-related gene *TaATG8j* to modulate cell death–associated defense responses (Mamun et al. 2018). Furthermore, genome-wide association studies (GWAS) have linked autophagy-related genes to stripe rust resistance, underscoring the pivotal role of autophagy in wheat immunity (Liu et al. 2015).

Among the core autophagy components, ATG6/Beclin1 serves as a central scaffold protein in the class III phosphatidylinositol-3-kinase (PI3K) complexes, which are essential for autophagosome initiation and endosomal trafficking (Xu et al. 2017). In *Arabidopsis*, ATG6/Beclin1 interacts with VPS34, VPS15, and either ATG14 or VPS38 to form functionally distinct complexes (Liu et al. 2020). Besides, ATG6/Beclin1 has been shown to restrict viral replication by mediating autophagic degradation, demonstrating its critical role in plant immunity (Li et al. 2018). Notably, TaATG6c, a wheat ATG6 homolog, is essential for autophagy biogenesis and contributes to immune responses against fungal pathogens, thereby linking autophagy to wheat defense mechanisms (Yue et al. 2015).

Hydrogen sulfide (H_2_S) is an important gaseous signaling molecule that regulates diverse plant physiological processes, including stress responses, hormone signaling, and autophagy, largely through protein persulfidation (Wang et al. 2021). Notably, the core autophagy protein ATG18a undergoes persulfidation, enhancing its role in autophagosome formation (Aroca et al. 2021). H_2_S has been shown to promote autophagy and reduce cell death under submergence stress by modulating ATG gene expression (Xuan et al. 2022) and to activate defense responses to limit pathogen-induced damage (Vojtovič et al. 2020). In rice, *Xanthomonas* infection triggers H_2_S production, leading to ROS bursts and activation of defense genes (Zhang et al. 2025), while exogenous H_2_S mitigates damage caused by *Pseudomonas syringae* pv. tomato DC3000 (Zhao et al. 2025). Despite these findings, the role of H_2_S in wheat resistance to stripe rust remains largely unexplored.

In this study, we demonstrate that H₂S enhances wheat resistance to stripe rust by activating autophagy. Comparative persulfidome analysis identified the autophagy regulator TaATG6c as a pathogen-responsive H₂S target, with persulfidation at Cys177 and Cys180 occurring specifically during *Pst* infection. Functional analyses using VIGS revealed that TaATG6c positively regulates wheat defense and is required for H₂S-induced resistance. Moreover, mutation of the persulfidated cysteine residues compromises TaATG6c-mediated resistance and autophagy activation. Collectively, these results indicate that H_2_S promotes autophagy during *Pst* infection by modulating TaATG6c activity through site-specific persulfidation, thereby enhancing wheat immunity. This work provides the first evidence that ATG6c is regulated by persulfidation in wheat and uncovers a previously unrecognized H₂S-dependent mechanism linking redox signaling, autophagy, and disease resistance.

## Result

### H₂S positively regulates wheat resistance to stripe rust

**Fig. 1:**
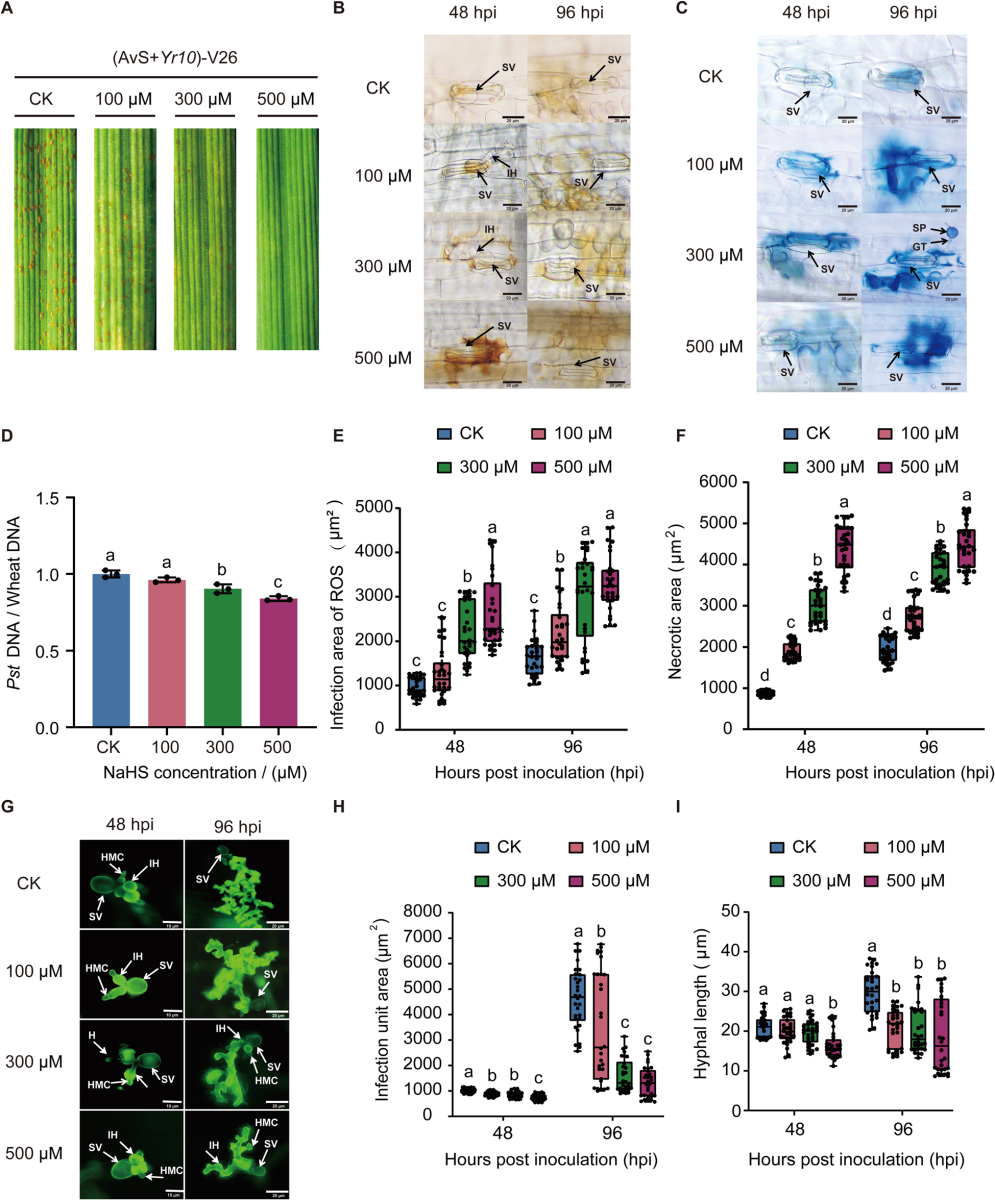
Positive role of H₂S in wheat resistance to *Pst*. **A** Representative phenotypes of *Triticum aestivum* (cv. AvS + *Yr10*) seedlings inoculated with *Pst* (V26) and treated with different concentrations of the hydrogen sulfide donor NaHS (0, 100, 300, and 500 μM) for 14 days. The fourth leaves of seedlings at the two-leaf stage were inoculated with V26, and phenotypes were recorded at 14 dpi. **B** Microscopic observation of ROS accumulation in wheat leaves following *Pst* inoculation with or without NaHS treatment. Wheat leaves were stained with DAB to visualize ROS accumulation at infection sites at 48 and 96 hpi. Scale bars, 20 μm. **C** Representative micrographs of hypersensitive response (HR) necrosis in wheat leaves following *Pst* inoculation with or without NaHS treatment. HR was visualized by trypan blue staining at 48 and 96 hpi. Scale bars, 20 μm. **D** Quantification of *Pst* biomass shown in **A**. **E** Quantification of ROS accumulation area shown in **B**. **F** Quantification of necrotic area shown in **C**. **G** Representative micrographs showing *Pst* infection structures in wheat leaves under different NaHS treatments. Fungal structures were stained with WGA-Alexa Fluor 488 and visualized by fluorescence microscopy at 48 and 96 hpi. Scale bars, 10 μm or 20 μm. **H** Quantification of infection unit area shown in **G**. **I** Quantification of fungal hyphal length shown in **G**. The data in **D** are the mean values ± SE (*n* = 3). The data in **B**, **C** and **E** to **I** are the mean values ± SE (*n* = 30). Within each group of experiments, there is a significant difference at the *P* < 0.05 level between the bars showing different letters (Duncan’s multiple range tests). Abbreviations: SV, substomatal vesicle; HMC, haustorial mother cell; IH, infection hyphae; H, haustorium; GT, germ tube; SP, spore

H₂S is a well-known signaling molecule involved in plant growth, development, H_2_S is a well-known signaling molecule involved in plant growth, development, and stress tolerance (Jaiswal et al. 2025). In our study, exogenous application of NaHS shortened the longest root length, promoted lateral root elongation, increased plant height, and enhanced dry matter accumulation (Fig. S1A–C). Consistent with its reported role in plant immunity (Vojtovič et al. 2020), H_2_S also enhanced wheat resistance to stripe rust. Wheat seedlings inoculated with *Pst* race V26 were sprayed with 100, 300, or 500 μM NaHS. Compared with untreated inoculated plants, NaHS-treated plants exhibited attenuated disease symptoms and reduced fungal proliferation, with the strongest effect observed at 500 μM (Fig. 1A and D). Consistently, exogenous application of 300 μM slow-releasing H_2_S donor GYY4137 suppressed pathogen development, whereas treatment with 300 μM H_2_S scavenger HTs in a non-compatible interaction increased necrotic lesion area (Fig. S2A-B). Quantitative analyses revealed that ROS accumulation and hypersensitive cell death area increased in a concentration-dependent manner, accompanied by a corresponding decrease in hyphal area and length (Fig. 1B, C, E-I). These results indicate that NaHS enhances wheat immunity against stripe rust in a dose-dependent manner.

### H₂S induces persulfidation of TaATG6c under stripe rust stress

**Fig. 2:**
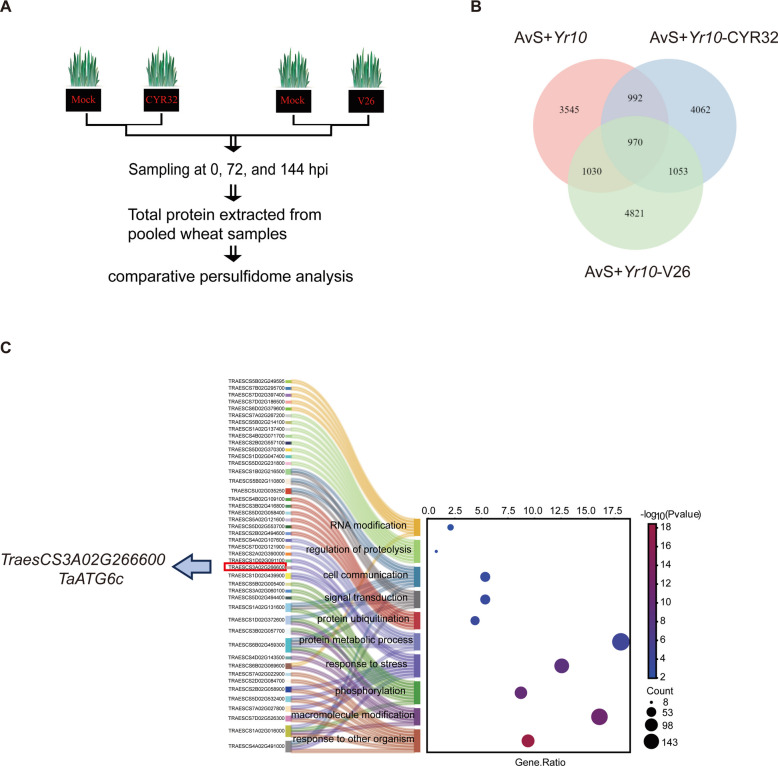
H₂S induces persulfidation of TaATG6c under stripe rust stress. **A** The workflow of persulfidome analysis to identify proteins involved in regulating resistance to *Pst*. **B** Venn diagram showing overlap of persulfidated proteins in (AvS + *Yr10*) wheat seedlings under mock treatment, or inoculated with *Pst* races CYR23 and V26. **C** GO analysis of persulfidated proteins commonly identified in (AvS + *Yr10*) wheat seedlings inoculated with both *Pst* races CYR23 and V26

H_2_S regulates diverse physiological processes in plants primarily through protein persulfidation (Aroca et al. 2018; Zhang et al. 2021). To determine whether the protective effect of H_2_S against stripe rust involves persulfidation, we performed a comparative persulfidome analysis of wheat leaves inoculated with *Pst* races CYR32/V26 and uninoculated controls (Fig. 2A). We identified 16,473 persulfidated proteins, among which 1,053 were uniquely modified in inoculated samples (Fig. 2B). GO enrichment analysis revealed that the proteins specifically persulfidated upon *Pst* infection were significantly enriched in biological processes associated with plant immunity and stress adaptation. As shown in Fig. 2C, the most prominent categories included response to other organism, response to stress, and signal transduction, reflecting the strong involvement of persulfidation in defense-associated pathways. In addition, processes related to macromolecule modification, phosphorylation, protein ubiquitination, and regulation of proteolysis were also significantly enriched. Notably, within the stress-response category, TaATG6c was the only protein that showed a direct functional association with the autophagy pathway (Supplementary Table S3). TaATG6c is an essential autophagy regulatory factor required for autophagy initiation and pathogen defense (Wang et al. 2018; Lai et al. 2011). The enrichment of TaATG6c in the stress-response category suggests that its persulfidation may contribute to the activation of autophagy during *Pst* infection. Collectively, these results indicate that H_2_S-mediated persulfidation preferentially targets proteins involved in stress signaling and protein homeostasis, thereby modulating wheat immune responses to *Pst*.

### Cys177 and Cys180 of TaATG6c undergo persulfidation

**Fig. 3:**
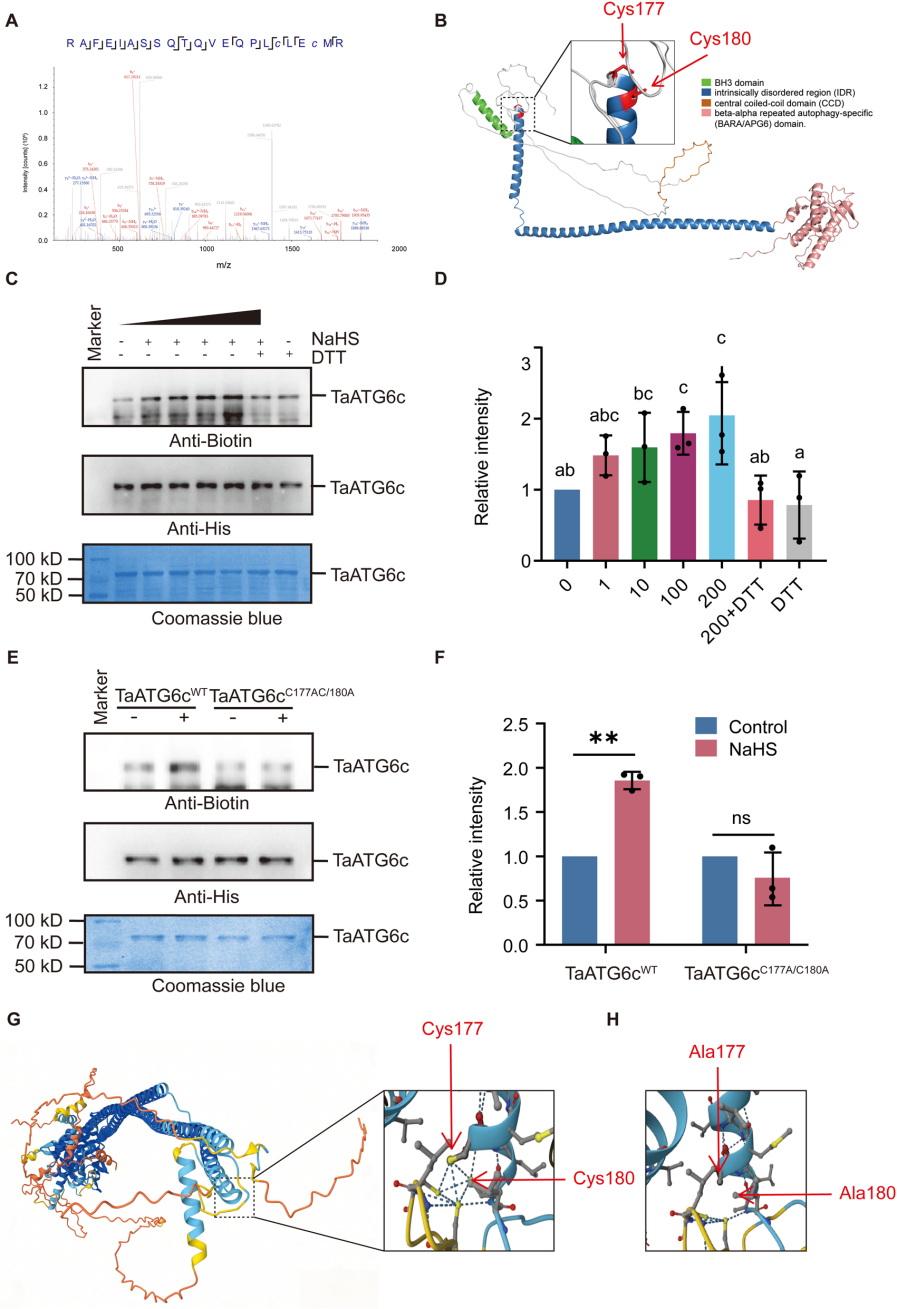
Cys177 and Cys180 of ATG6c undergo persulfidation. **A** Mass spectrometric analysis of TaATG6c. LC–MS/MS spectrum shows that the peptide, RAFEIASSQTQVEQPLc177LEc180MR is persulfidated. The fragment result shows that Cys177 and Cys180 are specifically persulfidated based on a mass shift of 31.97207 Da. **B** A 3D model of TaATG6c, Residues C177, C180 are shown in the model. **C** In vitro NaHS-induced persulfidation of TaATG6c was detected by using the biotin-switch assay. Proteins were incubated with the indicated con centration of NaHS or NaHS plus DTT (10 mM) for 30 min. **D** Quantification of persulfidation levels shown in **C**. The data are means ± SE (*n* = 3). Within each group of experiments, there is a significant difference at the P < 0.05 level between the bars showing different letters (Duncan’s multiple range tests). **E** In vitro persulfidation of TaATG6c C-to-A site-directed mutants was detected by using the biotin-switch assay. Proteins were treated with or without NaHS (200 mM) for 30 min. **F** Quantification of persulfidation levels shown in E. The data are means ± SE (*n* = 3). Within each set of experiments, bars with different asterisks are significantly different (Student’s *t*-test: *P < 0.05, **P < 0.01, ***P < 0.001). **G** AlphaFold-based structural modeling of TaATG6c (blue) and TaATG14 (orange). The interaction occurs through the coiled-coil domains of both proteins. The panel shows that wild-type TaATG6c forms a stronger multivalent interaction network with TaATG14. **H** The panel illustrates a disruption of hydrogen bonding at the TaATG6–TaATG14 interface following substitution of Cys177 and Cys180 with alanine in TaATG6c (TaATG6c^C177A/C180A^)

Comparative persulfidome analysis reveals TaATG6c, an autophagy-related protein essential for autophagy initiation, as a stress-responsive persulfidated protein. Two persulfidation sites, Cys177 and Cys180, were identified by mass spectrometry, each exhibiting a characteristic mass shift of 31.97207 Da (Fig. 3A). To explore the structural context of these sites, we constructed a three-dimensional (3D) model of wheat TaATG6c (Fig. 3B). The protein comprises four major domains: an N-terminal BH3 domain, an intrinsically disordered region (IDR), a central coiled-coil domain (CCD), and a C-terminal beta–alpha repeated autophagy-specific (BARA/APG6) domain (Wang et al. 2025). Cys177 and Cys180 are located within the CCD and exposed on the protein surface, suggesting that their modification may modulate protein–protein interactions or conformational dynamics during autophagy initiation. Persulfidation of TaATG6c was confirmed using the modified biotin-switch method (MBSM), in which anti-biotin immunoblotting reflects the degree of modification (Mustafa et al. 2009). NaHS treatment induced a concentration-dependent increase in TaATG6c persulfidation, whereas the reducing agent DTT markedly diminished the signal (Fig. 3C-D). Alanine substitution of Cys177 and Cys180 (TaATG6c^C177A/C180A^) completely abolished the NaHS-induced modification (Fig. 3E-F), confirming these residues as the critical persulfidation sites required for H_2_S-mediated regulation of TaATG6c. In contrast, MBSM revealed that TaATG6a and TaATG6b did not exhibit detectable persulfidation (Fig. S2A–B), supporting the specificity of TaATG6c as the H_2_S-responsive ATG6 paralog.

To evaluate the role of Cys177 and Cys180 in the interaction between TaATG6c and its autophagy partner ATG14 (Liu et al. 2020), alphaFold-based structural modeling was performed. The model indicated that both residues are positioned at the interaction interface, and that their mutation weakens TaATG6c–ATG14 binding, as evidenced by a reduction in hydrogen bonds at the interface (Fig. 3G-H). Subcellular localization analysis revealed that NaHS treatment did not alter the distribution of TaATG6c in *Nicotiana benthamiana* (Supplementary Fig. S3). This indicates that H_2_S-induced persulfidation regulates TaATG6c function primarily through modulation of its interaction properties rather than subcellular relocalization.

### TaATG6c positively regulates wheat resistance to stripe rust

**Fig. 4:**
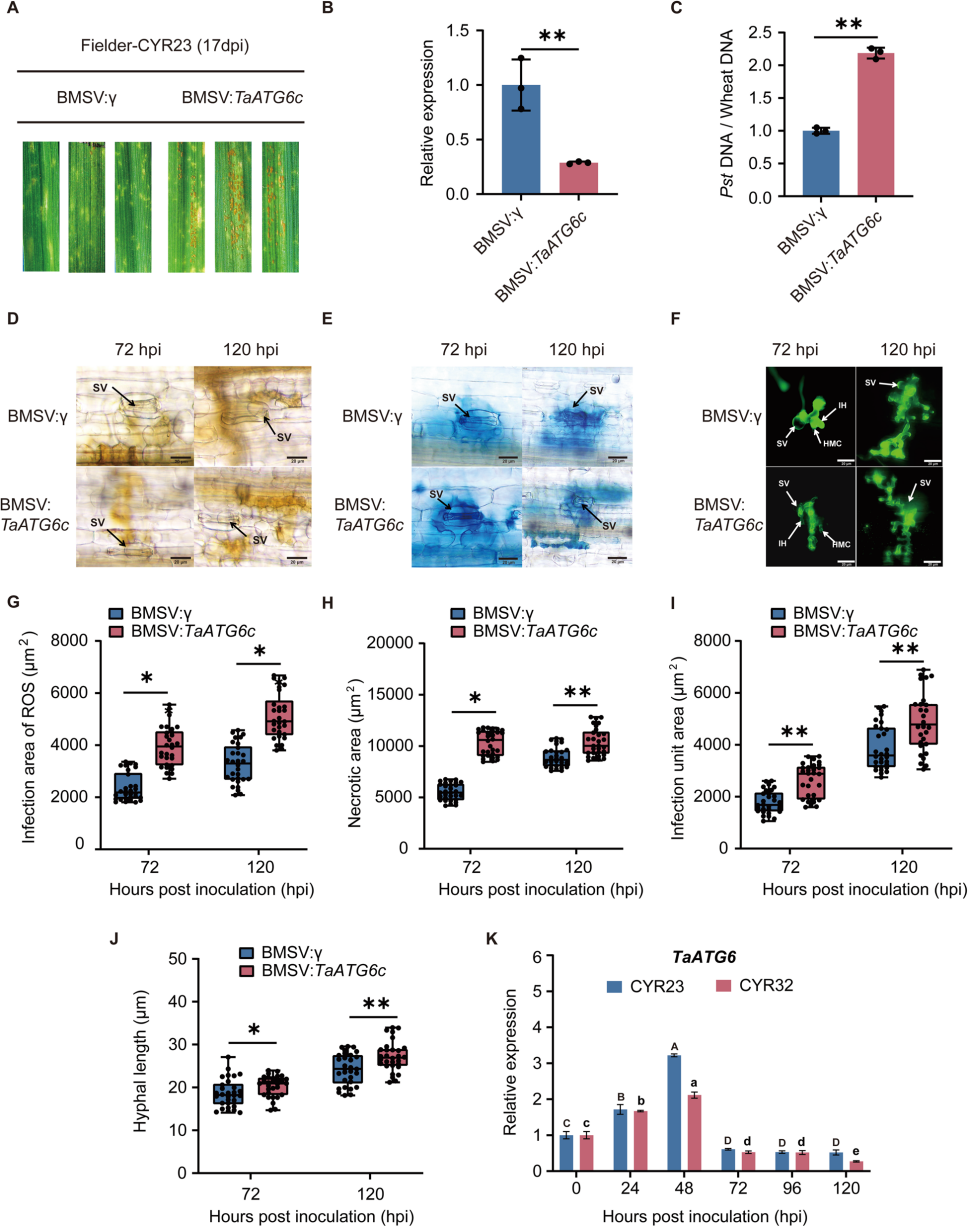
ATG6c Positively Regulates Wheat Resistance to Stripe Rust. **A** Representative leaves of silenced and control plants at 17 days post inoculation (dpi) with *Pst* race CYR23. Seedlings of Triticum aestivum cv. Fielder were pre-inoculated with either the BSMV:γ empty vector (EV) or BSMV:*TaATG6c* using Barley stripe mosaic virus (BSMV)-mediated virus-induced gene silencing (VIGS). **B** Gene expression level of *TaATG6c* in **A**. **C** Quantification of *Pst* biomass shown in **A**. **D** Microscopic observation of ROS accumulation in wheat leaves of silenced and control plants at 17 days post inoculation (dpi) with *Pst* race CYR23. Wheat leaves were stained with DAB to visualize ROS accumulation at infection sites at 72 and 120 hpi. Scale bars, 20 μm. **E** Representative micrographs of hypersensitive response (HR) necrosis in wheat leaves of silenced and control plants at 17 days post inoculation (dpi) with *Pst* race CYR23. HR was visualized by trypan blue staining at 48 and 96 hpi. Scale bars, 20 μm. **F** Representative micrographs showing *Pst* infection structures in wheat leaves of silenced and control plants at 17 days post inoculation (dpi) with *Pst* race CYR23. Fungal structures were stained with WGA-Alexa Fluor 488 and visualized by fluorescence microscopy at 48 and 96 hpi. Scale bars, 20 μm. **G** Quantification of ROS accumulation area shown in **D**. **H** Quantification of necrotic area shown in **E**. **I** Quantification of infection unit area shown in **F**. **J** Quantification of fungal hyphal length shown in **F**. **K** Gene expression of *TaATG6c* in wheat cv. Fielder leaves inoculated with *Pst* races CYR23 and CYR32. Leaf tissues were collected at 0, 24, 48, 72, 96, and 120 h post inoculation (hpi). The data are the mean values ± SE (*n* = 3). Within each group of experiments, there is a significant difference at the P < 0.05 level between the bars showing different letters (Duncan’s multiple range tests). Different uppercase letters **A-D** above bars denote significant differences among time points for CYR23, and different lowercase letters a-e denote significant differences among time points for CYR32. The data in **B** and **C** are the mean values ± SE (*n* = 3). The data in **D** to **J** are the mean values ± SE (*n* = 30). Within each set of experiments, bars with different asterisks are significantly different (Student’s *t*-test: *P < 0.05, **P < 0.01, ***P < 0.001). Abbreviations: SV, substomatal vesicle; HMC, haustorial mother cell; IH, infection hyphae; H, haustorium; GT, germ tube; SP, spore

Although TaATG6c is identified as a persulfidated protein, its functional role in wheat resistance to stripe rust remained unclear. To address this, we employed VIGS to suppress *TaATG6c* expression by targeting a conserved region shared by *TaATG6a*, *TaATG6b*, and *TaATG6c* due to their high sequence similarity, achieving an average silencing efficiency of approximately 70% (Fig. 4B). Upon inoculation with the avirulent race CYR23, *TaATG6*-silenced plants exhibited more severe disease symptoms compared with BSMV:γ controls (Fig. 4A), which was further supported by qPCR showing significantly higher *Pst* biomass in silenced leaves (Fig. 4C). Microscopic observations revealed that knockdown of *TaATG6* resulted in enlarged infection sites with increased hyphal growth and infection unit area (Fig. 4D–H). Interestingly, although silencing of *TaATG6* resulted in elevated ROS accumulation and enhanced cell death (Fig. 4G–H), these responses failed to effectively restrict fungal proliferation (Fig. 4I–J). Consistently, autophagy activator LiCl suppressed *Pst* growth, whereas autophagy inhibitor 3-MA significantly increased *Pst*–induced necrotic lesions on wheat leaves (Fig. S2A–B). Together, these results indicate that excessive ROS accumulation resulting from impaired autophagy is insufficient to limit pathogen proliferation. Furthermore, qRT-PCR analysis revealed that *TaATG6* transcript levels were significantly induced during *Pst* infection, peaking at 48 h post-inoculation (hpi). Expression was markedly higher in the incompatible (CYR23) interaction than in the compatible (CYR32) interaction (Fig. 4K).

### H_2_S promotes wheat resistance to stripe rust by regulating autophagy via ATG6c persulfidation

**Fig. 5:**
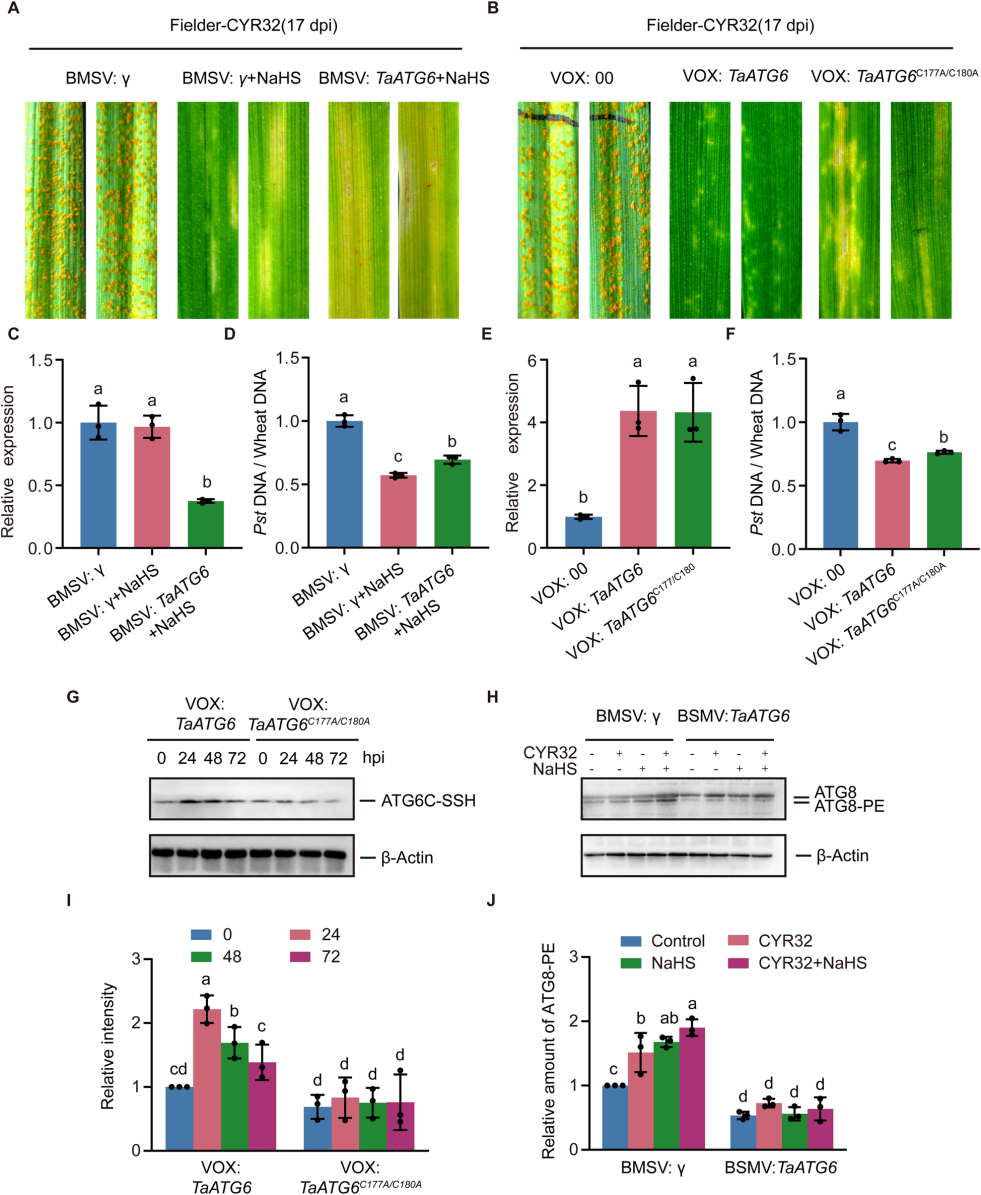
H_2_S Promotes Wheat Resistance to Stripe Rust by Regulating Autophagy via ATG6c Persulfidation. **A** Representative leaves of *TaATG6*-silenced and control wheat plants (cv. Fielder) at 17 dpi with *Pst* strain CYR32, with or without exogenous H₂S treatment. Barley stripe mosaic virus (BSMV)-mediated virus-induced gene silencing (VIGS) was used to pre-inoculate seedlings of the wheat field cultivar Fielder, with BSMV:γ empty vector (EV) or BSMV:*TaATG6* as the inoculation vectors. **B** Representative leaves of *TaATG6*-overexpressing and control wheat plants (cv. Fielder) at 17 dpi following inoculation with *Pst* strain CYR32. Barley stripe mosaic virus (BSMV)-mediated virus-induced gene overexpression (VOX) was used to pre-inoculate seedlings of wheat cultivar Fielder, with VOX:00 empty vector (EV), VOX:*TaATG6*, or VOX:*TaATG6*^C177A/C180A^ as the inoculation vectors. **C** Expression level of *TaATG6* in **A**. **D** Quantification of *Pst* biomass in **A**. **E** Expression level of *TaATG6* in **B**. **F** Quantification of *Pst* biomass in **B**. **G** In vivo *TaATG6* persulfidation analysis in *TaATG6*-overexpressing plants and *TaATG6* ^C177A/C180A^ -overexpressing plants, protein samples were collected at 0, 24, 48, and 72 hpi, Anti-HA antibody was used for immunoblotting. β-*Actin* was used as a loading control for the western-blot analysis. **H** ATG8 protein levels in the leaves of *TaATG6*-silenced and control wheat plants at 17 dpi with different treatments. Anti-ATG8 antibody was used for immunoblotting, β-*Actin* was used as a loading control for the western-blot analysis. **I** Quantification of persulfidation levels shown in **G**. The data are **J** Quantification of ATG8-PE accumulation shown in **H**. The data in C- F and I- J are means ± SE (n = 3). Within each group of experiments, there is a significant difference at the P < 0.05 level between the bars showing different letters (Duncan’s multiple range tests)

To determine whether H_2_S-enhanced resistance to stripe rust is mediated through TaATG6c-dependent autophagy, we first examined the effect of NaHS treatment on CYR32 infection in *TaATG6*-silenced plants. In control plants, NaHS treatment markedly alleviated disease symptoms caused by CYR32; however, this protective effect was substantially attenuated in *TaATG6*-silenced plants (Fig. 5A, 5C–D), indicating that TaATG6c is required for the full resistance-promoting effect of H_2_S. Transient overexpression of *TaATG6* significantly enhanced wheat resistance to CYR32, whereas mutation of the persulfidation sites (Cys177 and Cys180) markedly compromised this resistance enhancement (Fig. 5B, 5E–F). These results suggest that persulfidation of TaATG6c is critical for its resistance-associated function. To further investigate whether TaATG6 undergoes pathogen-responsive persulfidation. Endogenous biotin-switch assays were performed using wheat plants transiently expressing TaATG6. Protein samples were collected at 0, 24, 48, and 72 hpi. TaATG6 persulfidation increased rapidly following *Pst* inoculation, peaked at 24 hpi, followed by a gradual decline at 48 h and 72 h, although remaining higher than the basal level (Fig. 5G and I). In contrast, the cysteine mutant TaATG6^C177A/C180A^ displayed consistently low persulfidation levels and failed to show pathogen-induced changes over the same time course (Fig. 5G and I). These results demonstrate that TaATG6c undergoes dynamic persulfidation in response to *Pst* infection. To assess whether TaATG6c is required for autophagy activation during *Pst* infection and H_2_S treatment, we analyzed the accumulation of lipidated ATG8 (ATG8–PE), a marker of autophagosome formation. In the wild-type cultivar Fielder after virus inoculation, both CYR32 infection and NaHS treatment significantly increased ATG8–PE levels, and their combined treatment further enhanced ATG8–PE accumulation (Fig. 5H and J). In contrast, *TaATG6*-silenced plants exhibited a markedly reduced basal level of ATG8–PE, and neither CYR32 infection nor NaHS treatment effectively induced ATG8–PE accumulation (Fig. 5H and J). Taken together, these results indicate that H_2_S-mediated persulfidation of TaATG6c promotes wheat resistance to stripe rust by enabling effective activation of autophagy during *Pst* infection.

## Discussion

The regulatory role of H_2_S in various plant physiological processes has been well established (Zhang et al. 2022; Li et al. 2024; Jurado-Flores et al. 2023), yet its potential role and underlying mechanisms in wheat resistance to stripe rust remain unexplored. In this study, we demonstrate that H₂S promotes the growth of wheat seedlings under non-stress conditions (Fig. S1A-C), consistent with the findings of Dai et al. (2024) Previous studies have shown that H_2_S is induced by pathogens in plants and exhibits significant antimicrobial effects, supporting its role in plant defense against fungal pathogens (Bloem et al. 2004, 2012). Moreover, H₂S alleviates the damage caused by the bacterial pathogen *Pst* DC3000 in tomato (Zhao et al. 2025). We found that H_2_S prevented the proliferation of *Pst* and enhanced wheat immunity (Fig. 1A and D), representing the first demonstration that H_2_S enhances wheat resistance to stripe rust and extending its recognized role in plant defense.

H_2_S regulates plant growth and development through the persulfidation of proteins. Key proteins in the ABA signaling pathway, stomatal movement, and stress responses have been shown to undergo persulfidation (Zhou et al. 2021; Liu et al. 2025; Chen et al. 2020; Wang et al. 2021). In our comparative persulfidome analysis of *Pst*-infected wheat, we identified TaATG6c, an essential regulator of autophagy initiation, as a differentially persulfidated protein (Fig. 2C). Site-specific validation confirmed that Cys177 and Cys180 are persulfidated in response to H_2_S (Fig. 3A). Structural modeling revealed that both residues are located within the central coiled-coil domain (CCD) and exposed on the protein surface—a configuration typically involved in mediating protein–protein interactions within the autophagy initiation complex (Fig. 3B, Wang et al. 2025). This spatial positioning suggests that persulfidation at these sites could modulate TaATG6c’s interaction dynamics, thereby influencing autophagy activation during pathogen challenge, the model predicted by AlphaFold-based structural modeling also prove this (Fig. 3G and H). To our knowledge, this is the first evidence that persulfidation regulates autophagy in wheat, in line with reports from *Arabidopsis*, where persulfidation of Cys80 in ATG18a enhances its interaction with the autophagy machinery and promotes autophagosome formation (Aroca et al. 2021). Furthermore, proteins like ATG4a, ATG3, ATG5, and ATG7 have also been shown to undergo persulfidation (Laureano-Marín et al. 2020; Gotor et al. 2022; Aroca et al, 2022).

Autophagy is vital in plant immunity, particularly against pathogen invasion. Recent studies have revealed that autophagy enhances plant immunity (Jeon et al. 2025). Additionally, phosphorylation of the autophagy-related protein ATG18a has been shown to inhibit autophagy, weakening Arabidopsis resistance to pathogens (Zhang et al. 2021). These findings underscore the importance of post-translational regulation of autophagy-related proteins in disease resistance, aligning with our results linking H_2_S-induced persulfidation of TaATG6c to improved wheat resistance against stripe rust. Furthermore, VIGS assays demonstrated that silencing of *TaATG6c* resulted in increased *Pst* proliferation in wheat seedlings (Fig. 4C). Notably, although *TaATG6*-silenced plants accumulated higher levels of ROS, this did not translate into enhanced disease resistance (Fig. 4E). Since autophagy is known to play a key role in maintaining cellular ROS homeostasis, impaired autophagy likely reduces ROS-scavenging capacity, leading to more pronounced oxidative damage (Yamauchi et al., 2019; Oikawa et al., 2015). Our observations using the autophagy activator LiCl and the inhibitor 3-MA on wheat disease phenotypes further support this conclusion. Thus, in the absence of functional autophagy, ROS accumulation alone is insufficient to effectively restrict pathogen growth. Additionally, *TaATG6* expression was strongly induced upon *Pst* infection (Fig. 4K), supporting its positive regulatory role in wheat resistance to stripe rust. Similar functional relevance has been observed for *TaATG8j*, where gene silencing led to increased *Pst* biomass (Mamun et al. 2018), further supporting the crucial role of the autophagic process in plant immunity.

Through a combination of gene silencing, transient overexpression, and biochemical analyses (Fig. 5A-F), we systematically demonstrate that TaATG6c plays an indispensable role in H_2_S-mediated resistance to wheat stripe rust. As a core component of the class III phosphatidylinositol 3-kinase (PI3K) complex, ATG6 functions at the initiation stage of autophagosome formation, and its involvement in immunity-associated autophagy has been well documented in multiple plant–pathogen systems (Liu et al. 2005; Marshall and Vierstra 2018). Our results further showed that silencing of *TaATG6* markedly suppressed the accumulation of ATG8–PE (Fig. 5H and J), leading to impaired autophagy activation and enhanced pathogen proliferation. Importantly, NaHS treatment failed to restore autophagy induction and disease resistance in *TaATG6*-silenced plants (Fig. 5A and H), suggesting that H₂S promotes disease resistance mainly via TaATG6c, rather than through a broad or alternative autophagy-related mechanism.

Beyond its essential role in autophagy, the resistance-associated function of TaATG6c critically depends on cysteine-specific persulfidation. Although the overexpression construct was generated from a conserved coding region shared by *TaATG6a, TaATG6b,* and *TaATG6c*, both persulfidomic analysis and in vivo biotin-switch assays clearly demonstrate that persulfidation occurs specifically on TaATG6c. Moreover, mutation of the two key cysteine residues (Cys177 and Cys180) substantially compromised the resistance-enhancing effect mediated by ATG6c (Fig. 5B), supporting a model in which H_2_S fine-tunes ATG6c activity through site-specific post-translational modification rather than by simply altering protein abundance. Notably, TaATG6c persulfidation displayed a dynamic and pathogen-responsive pattern, underscoring its regulatory significance during infection (Fig. 5G and I). Protein persulfidation has been proposed as a rapid and reversible signaling mechanism that enables plants to coordinate defense responses within a precise temporal window (Aroca et al. 2018). Within this regulatory framework, TaATG6c likely functions as a redox-sensitive hub that integrates H_2_S signaling into the autophagy machinery, thereby ensuring timely and effective activation of autophagy under elevated pathogen pressure.

Taken together, our findings establish TaATG6c as a critical molecular link between H_2_S signaling and immune-related autophagy, and reveal persulfidation as a key post-translational mechanism underlying autophagy-mediated resistance to stripe rust in wheat. Specifically, H_2_S enhances wheat resistance by modulating autophagy through persulfidation of the autophagy-related protein TaATG6c, thereby providing mechanistic insight into how sulfur-based signaling contributes to plant disease resistance.

## Conclusion

In this study, we demonstrate that H₂S alleviates stripe rust disease in wheat and that the autophagy-related protein TaATG6c undergoes dynamic persulfidation in response to *Pst* infection. *TaATG6* expression is strongly induced upon pathogen challenge, and functional analyses establish TaATG6c as a positive regulator of wheat resistance to stripe rust. Moreover, both H₂S treatment and *Pst* infection promote the initiation of autophagy in a TaATG6c-dependent manner. Collectively, our findings uncover a previously unrecognized molecular mechanism in which H₂S signaling enhances wheat disease resistance by promoting autophagy initiation through persulfidation of TaATG6c, thereby strengthening the plant’s capacity to restrict *Pst* invasion.

## Method and materials

### Plant material and growth conditions

Wheat seedlings (*Triticum aestivum* L. cv. AvS + *Yr10*) were used in this study. This cultivar exhibits an incompatible interaction with the wheat stripe rust isolate CYR32, but a compatible interaction with isolate V26(Wu et al. 2023).Seeds were rinsed with deionized water (2–3 times), sterilized in 0.3% H₂O₂ for 24 h, and then soaked in fresh water. Germinated seeds with coleoptiles 1–2 cm in length were transferred to 96-well hydroponic trays containing 1 L Hoagland nutrient solution. Seedlings were grown in a controlled chamber at 16 °C/13 °C (day/night) with a 16 h light/8 h dark photoperiod.

### Pst inoculation and chemical treatments

Wheat seedlings at the two-leaf stage were used. Fresh urediniospores of *Pst* race V26 were mixed with isooctadecane (IHD) to form an orange-red spore suspension. The suspension was sprayed onto leaves from four directions using an air pump and spray gun at ~ 2.0 pressure, producing a fine mist for uniform coverage. After the leaf surface dried, plants were kept in the dark with misting for 48 h, then transferred to a growth chamber for further cultivation. All wheat seedlings inoculated with V26 were subjected to root irrigation with 100 µM, 300 µM, or 500 µM NaHS for 14 days, or left untreated.

### Histochemical analysis

Tissue samples were examined under an Olympus BX-51 fluorescence microscope (OLYMPUS, Tokyo, Japan). ROS accumulation in necrotic cells was visualized using 3,3'-diaminobenzidine (DAB) staining. Hypersensitive response (HR) zones in necrotic cells were observed by combining autofluorescence with trypan blue staining. For DAB staining, infected wheat leaves were incubated in 1 mg/mL DAB solution under strong light for 3–4 h, followed by decolorization with a bleaching solution (anhydrous ethanol: acetic acid, 1:1). According to previously described methods (Wang et al. 2017), *Puccinia striiformis* infection structures in exogenously treated and control plants were stained using wheat germ agglutinin (WGA) conjugated with Alexa Fluor 488 (Sigma-Aldrich, St. Louis, MO, USA).

### Quantification of Pst biomass by qRT-PCR

DNA was rapidly extracted from 14 dpi samples using the cetyltrimethylammonium bromide (CTAB) method. Quantitative real-time PCR (qRT-PCR) was performed using primers specific for *TaEF* and *PsEF,* Quantitative primers were designed by Premier 5.0 software and all primers were listed in Supplementary Table S1. Copy numbers of *TaEF* and *PsEF* were calculated from Ct values using standard curves, and the *PsEF/TaEF* ratio was determined. This ratio was compared to that of control plants to assess the extent of *Puccinia striiformis* colonization in wheat.

### Comparative persulfidome analysis

Total proteins were extracted from wheat samples subjected to different treatments using a commercial protein extraction kit (Beyotime, Shanghai, China), equal amounts of protein from each sample were subjected to liquid chromatography–tandem mass spectrometry (LC–MS/MS) for proteomic analysis. The samples were alkylated by incubating in 50 mM iodoacetamide (Sigma-Aldrich, St. Louis, MO, USA) for 40 min in the dark. Then the samples were digested with trypsin (Promega, Madison, WI, USA) enzyme at a mass ratio of 1: 50 for 16 h at 37 °C. Digested peptides were dissolved in a sample solution (0.1% formic acid and 2% acetonitrile). After centrifugation, the supernatant was analyzed using liquid chromatography–tandem mass spectrometry (LC–MS/MS) (Orbitrap Fusion Lumos; Thermo Fisher, United States). The results were analyzed using THERMO PROTEOME DISCOVER v.2.2.

### Gene ontology analysis

Differentially persulfidated proteins identified between the compatible and incompatible interaction systems (in comparison to the Mock group) were selected. Gene Ontology (GO) enrichment analysis of biological processes was performed using the Microbioinformatics online platform, a cloud-based platform for bioinformatic analysis and visualization (http://www.bioinformatics.com.cn/). Taking P-values ≤ 0.05 as the threshold. The target protein TaATG6c, which is involved in the "response to stress" process, was subsequently selected.

### Recombinant protein expression and site-directed mutagenesis

The complete coding regions of *TaATG6c* (TraesCS3A02G266600) were inserted in-frame into the plasmids pClod-SUMO (Invitrogen) using the ClonExpress II One Step Cloning Kit (Takara, Beijing, China). Primers were designed by Premier 5.0 software and all primers were listed in Supplementary Table S1. *Escherichia coli* BL21 cells expressed the recombinant proteins that were purified using HIS-tagged. Site-directed mutagenesis was performed using the Quick e II Site-Directed Mutagenesis Kit (Stratagene, Garden Grove, CA, USA).

### Subcellular localization of TaATG6cWT and TaATG6c^C177A/C180A^ in tobacco

Design primers with 15–20 bp homologous arms and a (*SpeI*) restriction site using Primer 5.0 software. Perform single digestion of the pBinGFP2.0 plasmid with SpeI to prepare a linearized vector. Ligate the amplified gene fragment with the linearized vector, followed by E. coli transformation. Submit samples for company sequencing, and select correct single colonies for culture and plasmid extraction. Plasmids were transformed into Agrobacterium tumefaciens, and single colonies were cultured to an OD₆₀₀ of 0.6. The bacterial suspension was infiltrated into *Nicotiana benthamiana* leaves. After 24 h, subcellular localization was observed using a fluorescence microscope, and images were captured with CellSens Entry software (Olympus, Japan).

### BSMV-mediated transient gene overexpression

Wheat cultivar Fielder was used as the experimental material. Conserved regions of approximately 500 bp of *TaATG6* and its mutant *TaATG6*^C177A/C180A^ were cloned into the PcaBS-γ-p2A-gene-HA vector. In vitro transcripts of the three-part BSMV genome were synthesized and inoculated into Fielder seedlings at the two-leaf stage, following the method of Holzberg et al. (2002). The empty BSMV vector (BSMV:00) served as a negative control. Fourteen days post-BSMV inoculation (dpi), the fourth leaf was challenged with *Pst* strain CYR32, and phenotypes were recorded from 14 to 17 dpi. All experiments were repeated at least three times.

### Immunochemical detection of persulfidated TaATG6c^WT^ and TaATG6c^C177A/C180A^

For in vitro persulfidation assays, Persulfidated proteins were detected using a modified biotin switch method (Mustafa et al. 2009). The purified recombinant proteins were treated with the indicated concentration of NaHS to increase the concentration of persulfidated protein or with 10 mM DTT to reduce all of the disulfide bonds; both treatments were carried out at 4 °C for 20 min. Residual NaHS was removed by Micro BioSpinP6 columns. The nontreated proteins were used as a control. The free-SH on the protein was first blocked with MMTS for alkylation, and then, the protein sample was reacted with biotinlabel to detect the persulfidated modification by an anti-biotin antibody (HRP Streptavidin; Sigma-Aldrich, St. Louis, MO, USA). The total proteins were detected by immunoblotting using an anti-HIS antibody (Abbkine, Wuhan, China). For in vivo persulfidation assays. Wheat leaves transiently overexpressing *TaATG6* and *TaATG6*^*C177A/C180A*^ were used to extract protein. After biotin switch, persulfidated proteins were affinity-captured on immobilized streptavidin beads (Thermo Fisher Scientific, Waltham, USA). The levels of persulfidated TaATG6 and TaATG6^C177A/C180A^ were determined by immunoblotting using the anti-HA antibody (Beyotime, Shanghai, China).

### BSMV-mediated gene silencing

To silence the candidate gene *TaATG6c*, wheat cultivar Fielder was used as the experimental material. A conserved region of about 200 bp was cloned into the γ-PCR vector to generate the silencing construct. In vitro transcripts of the BSMV tripartite genome were synthesized and inoculated onto Fielder seedlings at the two-leaf stage, following the method of Holzberg et al. (2002). The empty BSMV vector (BSMV:00) was used as the negative control. At 14 dpi with BSMV, the fourth leaves were inoculated with *Pst* race CYR23, and phenotypes were recorded from 14 to 17 dpi.. All experiments were repeated at least three times.

### Protein extraction and western blotting

For protein extraction, total proteins were extracted from wheat samples subjected to different treatments using a commercial protein extraction kit (Beyotime, Shanghai, China), the extracted proteins were heated at 95 °C for 15 min; this was followed by separation using 10% SDS-PAGE. Detection of ATG8 was performed according to the method described by Li et al. (2014), with minor modifications. Briefly, the denatured proteins were separated on a 15% SDS-PAGE gel in the presence of 6 M urea. For western blotting, the proteins on the SDS-PAGE gel were transferred to a polyvinylidene fluoride membrane. Then, the membrane was blocked for 1 h in TBST buffer (20 mM Tris, pH 7.5, 150 mM NaCl, and 0.1% Tween 20) with 5% skim milk powder at room temperature and then incubated for 1 h in TBST buffer with 1% BSA containing a rabbit anti-Atg8 polyclonal antibody (Agrisera, Vännäs, Sweden), mouse anti-actin polyclonal antibody (Abbkine, Wuhan, China). After incubation with a goat anti-mouse horseradish peroxidase-linked anti body (Abbkine, Wuhan, China) or goat anti-rabbit horseradish peroxidase-linked antibody (Abbkine, Wuhan, China), the complexes on the blot were visualized using the SuperSignal West Pico Chemiluminescent Substrate (Thermo Fisher Scientific, Waltham, USA) by following the manufacturer’s instructions.

### Gene expression analysis

Total RNA was extracted from wheat samples rapidly frozen and ground in liquid nitrogen, followed by reverse transcription into cDNA. qRT-PCR was performed using gene-specific primers for *TaATG6c*, with *NACT* (actin) serving as the internal reference. Each time point included at least three independent biological replicates. Relative gene expression levels were calculated using the 2^−ΔΔCT^ method.(Livak and Schmittgen 2001).

### Statistical analysis

Data obtained from experiments were statistically analyzed using SPSS 17.0 (https://www.ibm.com/cn-zh/spss). For comparisons among multiple groups, Duncan’s multiple range test was applied. A significance level of *P* < *0.05* was used for all statistical analyses.

## Supplementary Information

Supplementary Material 1. Figure S1. Exogenous H₂S promotes wheat seedling growth under non-stress conditions. A Representative images of wheat seedlings (AvS+Yr10) treated with 0 (CK), 100, 300, and 500 μM NaHS for 14 days. B–J Morphological and physiological parameters of seedlings under different NaHS concentrations: B plant height; C shoot fresh weight per plant; D shoot dry weight per plant; E leaf phenotype; F root morphology; G maximum root length; H root number; I root dry weight per plant; and J root fresh weight per plant. Within each set of experiments, The data are the mean values ± SE (*n* = 30), bars with different asterisks are significantly different (Student’s *t*-test: *P < 0.05, **P < 0.01, ***P < 0.001).

Supplementary Material 2. Figure S2. Phenotypes of wheat resistance to stripe rust under different treatments. A Representative phenotypes of two-leaf-stage wheat seedlings (cultivar AvS+Yr10) at 15 days post-inoculation (dpi) with Pst V26 after treatment with 300 µM hydrogen sulfide donor NaHS, the slow-release donor GYY4137 (300 µM), or the autophagy activator LiCl (50 mM). B Representative phenotypes of two-leaf-stage wheat seedlings (cultivar AvS+Yr10) at 15 dpi with Pst CYR32 after treatment with the hydrogen sulfide scavenger HT (300 µM) or the autophagy inhibitor 3-methyladenine (3-MA; 5 mM).

Supplementary Material 3. Figure S3. TaATG6a and TaATG6b do not undergo persulfidation. A Biotin-switch assay to detect persulfidation of TaATG6a. Proteins were incubated with the indicated con centration of NaHS or NaHS plus DTT (10 mM) for 30 min. B Quantification of persulfidation levels shown in A. C Biotin-switch assay to detect persulfidation of TaATG6b. Proteins were incubated with the indicated con centration of NaHS or NaHS plus DTT (10 mM) for 30 min. D Quantification of persulfidation levels shown in C. The data in B and D are means ± SE (n = 3). Within each group of experiments, there is a significant difference at the P < 0.05 level between the bars showing different letters (Duncan’s multiple range tests)

Supplementary Material 4. Figure S4. Subcellular localization of TaATG6c and TaATG6cC177A/C180A. A Subcellular localization of TaATG6c and TaATG6cC177A/C180A in Nicotiana benthamiana under control conditions. Bars = 20 μm. B Subcellular localization of TaATG6c and TaATG6cC177A/C180A in Nicotiana benthamiana following exogenous H₂S application. Bars = 20 µm.

Supplementary Material 5. Figure S5. ATG6 protein levels in transiently overexpressing and control plants.

Supplementary Material 6. Figure S6. Alignment of Homologous Gene Sequences. A Graphical depiction of the coding sequence (CDS) for TaATG6c, a wheat homologue of TaATG6c. The sequence was sourced from the WheatOmics database (http://wheatomics.sdau.edu.cn/). The fragment indicated in red was used for Virus-Induced Gene Silencing (VIGS).The underlined segment is used for virus-induced gene overexpression experiments. B Multiple sequence alignment of the TaATG6c gene with its homologous sequences. The analysis includes the nucleotide sequences of TaATG6a, TaATG6b, and TaATG6c, which are located on the homologous group 3 chromosomes (3DL, 3BL, and 3AL, respectively) in the genome of hexaploid wheat. The alignment was performed with DNAMAN, and yellow shading indicates identical nucleotides across all sequences.

Supplementary Material 7. Table S1. Primers used for the functional analysis of TaATG6c.

Supplementary Material 8. Table S2. Persulfidated proteins identified under mock, CYR32, and V26 treatments.

Supplementary Material 9. Table S3. “Response to stress” pathway proteins.

## Data Availability

All data and materials are available in the paper and online supplemental files.

## Abbreviations

H₂S: Hydrogen sulfide
GO: Gene Ontology
GWAS: Genome-wide association studies
IDR: Intrinsically disordered region
CCD: Central coiled-coil domain
MBSM: Modified biotin-switch method
WGA: Wheat germ agglutinin
SV: Substomatal vesicle
IH: Infection hyphal
HMC: Haustorial mother cells
ROS: Reactive oxygen species
qRT-PCR: Quantitative reverse transcription-PCR
HR: Hypersensitive response
Hpi: Hours post inoculation
PCD: Programmed cell death
BSMV-VIGS: Barley stripe mosaic virus-induced gene silencing
LC–MS/MS: Liquid chromatography–tandem mass spectrometry
CTAB: Cetyltrimethylammonium bromide
