# Supplementary figures and images for: Hydrogen sulfide promotes wheat immunity against stripe rust through TaATG6c persulfidation
Source: Stress Biol. 2026 Feb 13;6(1):16. doi: 10.1007/s44154-026-00292-7 (PMC12905053; doi:10.1007/s44154-026-00292-7)

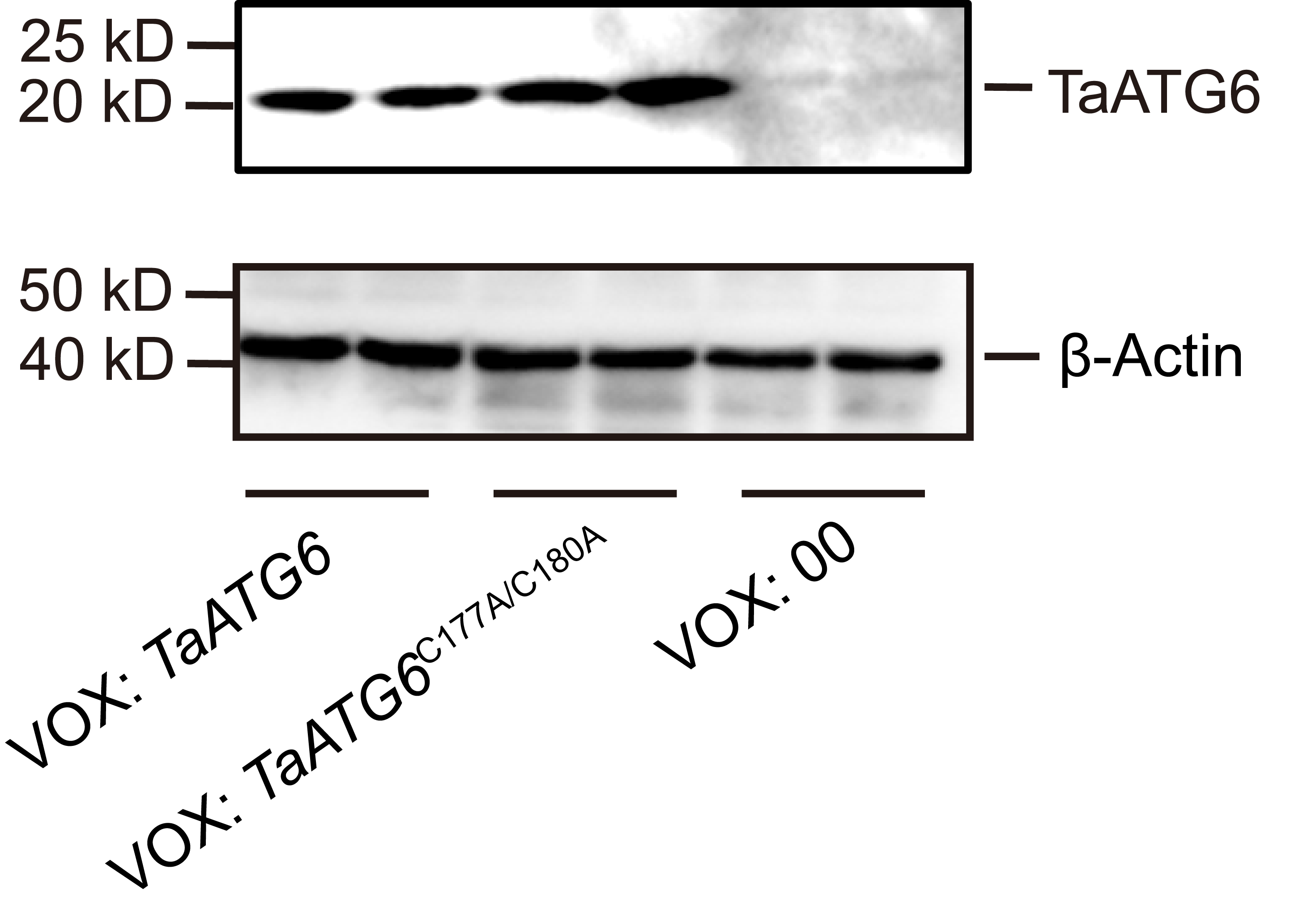

Supplement: Supplementary file 5 — Supplementary Material 5. Figure S5. ATG6 protein levels in transiently overexpressing and control plants. [file 44154_2026_292_MOESM5_ESM.tif]

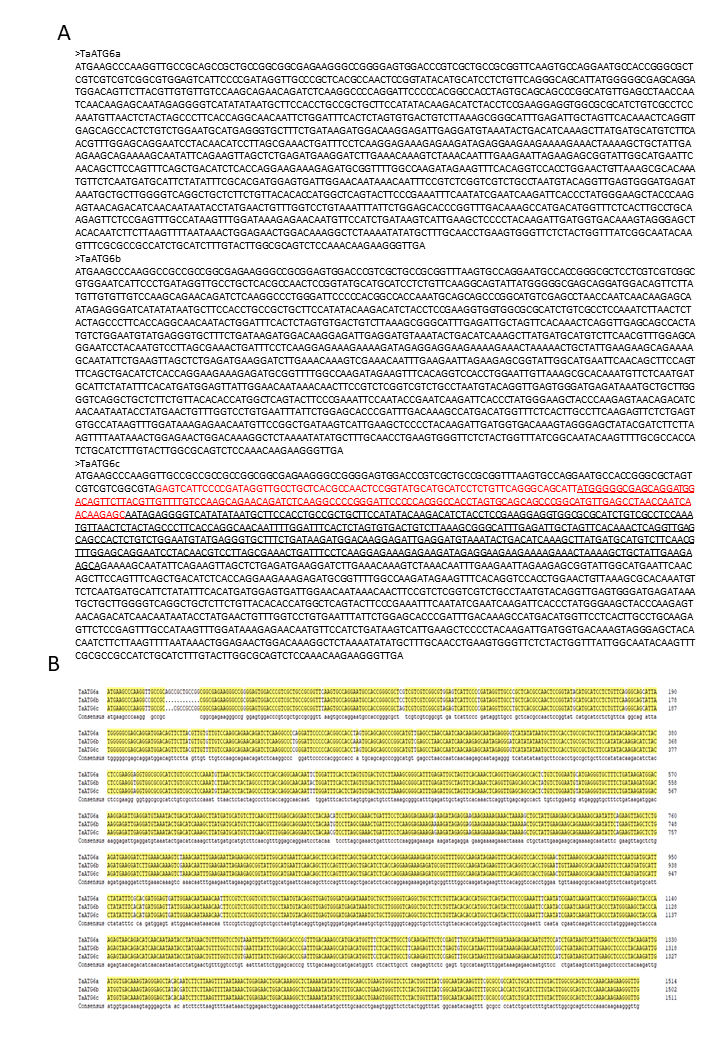

Supplement: Supplementary file 6 — Supplementary Material 6. Figure S6. Alignment of Homologous Gene Sequences. A Graphical depiction of the coding sequence (CDS) for TaATG6c, a wheat homologue of TaATG6c. The sequence was sourced from the WheatOmics database (http://wheatomics.sdau.edu.cn/). The fragment indicated in red was used for Virus-Induced Gene Silencing (VIGS).The underlined segment is used for virus-induced gene overexpression experiments. B Multiple sequence alignment of the TaATG6c gene with its homologous sequences. The analysis includes the nucleotide sequences of TaATG6a, TaATG6b, and TaATG6c, which are located on the homologous group 3 chromosomes (3DL, 3BL, and 3AL, respectively) in the genome of hexaploid wheat. The alignment was performed with DNAMAN, and yellow shading indicates identical nucleotides across all sequences. [file 44154_2026_292_MOESM6_ESM.tif]
